# A Predictive Model for Acute Respiratory Distress Syndrome Mortality Using Red Cell Distribution Width

**DOI:** 10.1155/2020/3832683

**Published:** 2020-01-04

**Authors:** Ala Alkhatib, Lori Lyn Price, Rania Esteitie, Peter LaCamera

**Affiliations:** ^1^Department of Medicine, Section of Pulmonary Diseases, Critical Care and Environmental Medicine, Tulane University Health Science Center, New Orleans, LA, USA; ^2^Tufts Clinical and Translational Science Institute, Tufts University, Boston, MA, USA; ^3^The Institute for Clinical Research and Health Policy Study, Tufts Medical Center, Boston, MA, USA; ^4^Department of Pulmonary and Critical Care, Covenant Healthcare, Saginaw, MI, USA; ^5^Department of Pulmonary and Critical Care, St. Elizabeth's Medical Center, Boston, MA, USA

## Abstract

**Methods:**

This observational retrospective cohort study includes 318 ARDS patients extracted from an ICU database between the years of 2001 and 2008. Clinical factors including age, gender, comorbidity score, Sequential Organ Failure Assessment (SOFA) score, and PaO_2_/FiO_2_ ratio were chosen for the base model to predict ICU mortality. The RDW value at the time of ARDS diagnosis was added to the base model to determine if it improved its predictive ability.

**Results:**

318 subjects were included; 113 (36%) died in the ICU. AUC for the base model without RDW was 0.76, and 0.78 following the addition of RDW [*p*=0.048]. The NRI was 0.46 (*p*=0.001), indicating that, in 46% of patients, the predictive probability of the model was improved by the inclusion of RDW.

**Conclusions:**

Adding RDW at time of ARDS diagnosis improved discrimination in a model using 4 clinical factors to predict ICU mortality.

## 1. Introduction

Acute Respiratory Distress Syndrome (ARDS) is an acute inflammatory response of the lung to stressful pulmonary or extrapulmonary processes [1]. An ability to reliably predict unfavorable outcomes in ARDS would greatly aid in the challenges of communicating clearly with families when discussing prognosis and goals of care and proper subject enrollment in clinical trials. Because of the known high mortality rate of ARDS, many studies have examined risk factors associated with severity and mortality. These efforts have identified age, comorbidities, presence of multiorgan dysfunction, and lung disease severity as factors correlating with ARDS outcomes [2–4].

Red blood cell distribution width (RDW) is a routinely available component of the complete blood count that reflects the size variability of red blood cells. RDW is calculated by dividing the standard deviation of red blood cell volume (measured by hematological analyzer) by the mean cell volume and multiplying by 100 to convert the result into a percentage. Higher RDW values indicate more variation in red blood cell volume [5]. Over the last few years, numerous studies have shown a relationship between higher RDW values and worse outcomes in different clinical conditions including coronary artery disease, acute kidney injury, and chronic obstructive lung disease [6–8]. Additionally, recent studies have established an association between RDW and mortality in critically ill patients [9]. An examination of whether RDW is predictive of ARDS outcomes has not been performed. Due to its availability in clinical settings, RDW values are attractive candidates for providing additional prognostic information about those with ARDS.

Therefore, the aim of this study was to first create a predictive model of ARDS mortality using demographic and clinical data previously described as correlating with ARDS outcomes. The statistical value of adding initial RDW results to the model was then examined. Additionally, we explored the relationship during the ICU course between RDW trends and ARDS outcomes.

## 2. Materials and Methods

### 2.1. Study Design and Database

As a part of this observational, retrospective cohort study, admissions to the intensive care units (ICUs) at Beth Israel Deaconess Medical Center (BIDMC) between the years of 2001-08 were reviewed through the Multiparameter Intelligent Monitoring in Intensive Care II (MIMIC-II) database [10, 11]. MIMIC-II is a publicly available database of prospectively collected data for all admissions to the intensive care units at BIDMC between the years of 2001 and 2008 and consists of 25,328 patients. This database which has been previously deidentified includes detailed information including vital signs, laboratory data, ventilator settings, severity of illness scores, radiology reports, and discharge notes of all patients. Survival during and after discharge is recorded by integrating MIMIC-II with the Social Security database. The data in MIMIC-II were approved by the institutional review boards of the Massachusetts Institute of Technology (No. 0403000206) and Beth Israel Deaconess Medical Center (2001-P-001699/14) for research.

### 2.2. Definition of ARDS

We used the Berlin criteria for ARDS in our study which includes (1) onset of the symptoms within one week of a known clinical insult or new or worsening respiratory symptoms, (2) bilateral opacities–not fully explained by effusions, lobar/lung collage, or nodules, and (3) respiratory failure not fully explained by cardiac failure of fluid overload. The Berlin criteria also use PaO_2_/FiO_2_ while receiving a minimum level of PEEP 5 cm H_2_O to stage ARDS severity as mild, moderate, and severe [12].

### 2.3. Inclusion and Exclusion Criteria

We reviewed the discharge summaries of all adult patients admitted to the medical ICU between January 2001 and December 2008 by searching for “Acute Respiratory Distress Syndrome,” “ARDS,” or “Acute Lung Injury,” and then evaluated the charts identified to determine whether Berlin criteria for ARDS were met and mechanical ventilation was used. To confirm that the criteria were met, the first PaO_2_/FiO_2_ ratio available was used and radiology reports and admission notes were reviewed. Our exclusion criteria included age <18 years and any possible alternative cause of elevated RDW values including a documented history of hematological malignancy, nutritional anemia, and blood transfusions during the same admission. All patients who met our inclusion and exclusion criteria were included in the study. RDW values were collected from routine complete blood count (CBC) results for all the patients who met our inclusion criteria from the time of admission to the time of discharge. Other data collected included age, major comorbidities, the Sequential Organ Failure Assessment (SOFA) score, and date of death if applicable. There were no additional inclusion or exclusion criteria.

### 2.4. Predictors and Outcome

The purpose of this study was to create a predictive model for mortality in ARDS using clinical factors and to determine whether the addition of RDW to the model improved its accuracy. Clinical factors including age, comorbidity score, Sequential Organ Failure Assessment (SOFA) score, and PaO_2_/FiO_2_ ratio were chosen for the base model. The primary outcome of our model was ICU mortality. Other secondary outcomes included in-hospital and 90-day mortality. Whether the trajectory of RDW changes during the course of ARDS was associated with in-hospital mortality was also explored.

### 2.5. Statistical Analysis

Means and frequencies were generated to describe our sample. Separate logistic regression models were performed for the primary outcome of ICU mortality and secondary outcomes of in-hospital mortality and 90-day mortality. For each outcome, we built two models. The base model included the risk factors age, sex, number of comorbid conditions, SOFA score, and first documented PaO_2_/FiO_2_ after diagnosis. The enhanced model was the base model plus the closest RDW to the time of diagnosis. Restrictive cubic splines were generated to test for linearity of the continuous variables and the logit. Several metrics were used to assess the benefit of adding RDW to the base model: (1) area under the receiver-operating characteristics curve (AUC), (2) net reclassification improvement (NRI), and (3) integrated discrimination improvement (IDI). NRI is calculated by adding the percent of patients who died that had an increased probability of death in the enhanced model compared to the base model to the percent of patients who survived that showed a reduction in predicted probability of death in the enhanced model. IDI is calculated by adding the average increase in predicted probability of death in patients who died to the average decrease in predicted probabilities of those who survived when comparing the base model to the enhanced model.

Mixed models with random intercepts and slopes were used to define the trajectories of RDW over time. The outcome was the RDW value and the independent variables were time and time squared, where time is the number of days between ARDS diagnosis and each RDW measurement. A slope was generated for each patient who had at least 4 RDW measurements. For each patient, if the quadratic term was statistically significant, the trajectory was considered concave (negative slope) or convex (positive slope). If the quadratic term was not significant but the main effect term was significant, the patient was classified as upward (positive slope) or downward (negative slope). Patients whose regression results yielded nonsignificant *p*-values for both the quadratic and main effects were considered to have a stable RDW trajectory. Chi-square tests and Fisher Exact tests were used to test for an association between trajectory category and mortality.

## 3. Results

We included a total of 318 patients from the MIMIC-II database into our study. The mean age of the patients was 58.6 years, and 52% were males. Baseline characteristics of the study population based on the ICU survival status are presented in Table 1. Mean (standard deviation) RDW value measured at the time closest to initial ARDS diagnosis was 15.3% (±2.3%). The ICU mortality for ARDS patients included in our study was 35.5%, while in-hospital mortality was 44.0%, closely resembling previously reported overall mortality rates of 43% [13]. Mortality rates were higher in male patients (40%) in comparison with female patients (31%). Mean age of patients who survived was 54 years, which was lower than the mean age of 67 years of those that died in the ICU.

The base model consisting of age, sex, comorbidity score, Sequential Organ Failure Assessment (SOFA) score, and PaO_2_/FiO_2_ and the base model plus RDW for the outcome of ICU mortality are presented in Table 2. Comparisons of the discriminative and predictive abilities of the 2 models are presented in Table 3 for all mortality outcomes.

The predictive accuracy of the new model with RDW for ICU mortality improved compared with the base model; AUC for the base model without RDW was 0.76, and 0.78 when RDW was added [*p*=0.048]. The net reclassification improvement (NRI) was 0.46 (*p*=0.001), indicating that 46% of the patients had improved predictive probabilities in the correct direction following the addition of RDW. The discrimination improvement (IDI) was 0.03 (*p*=0.005), which indicates that the model with RDW had slightly improved predictions for both patients who died and survived. We additionally evaluated other secondary outcomes including in-hospital mortality and 90-day mortality by using the same base and modified models. Adding RDW to the base model improved the AUC from 0.75 to 0.78 (*p*=**0.02**) in predicting in-hospital mortality and improved AUC from 0.76 to 0.79 (*p*=**0.01**) in predicting 90-day mortality. NRI and IDI for both outcomes also indicated improved predictive ability.

We also investigated whether RDW trends during the hospital course is associated with ARDS mortality. For this portion of the study, patients with less than 4 available RDW values were excluded from analysis. We categorized the remaining 272 patients into groups described as uptrending, downtrending, concave, convex, and no trend depending on the pattern or shape of their RDW results. Most of the patients (174 (64%)) had no trend in their RDW value; other patients were distributed into the remaining four groups as follows: 19 (7%) uptrending, 24 (9%) downtrending, 26 (10%) concave, and 29 (11%) convex (Table 4). The differences between mortality in the five different trends were evaluated using the fisher exact probability test with a *p*=0.33. However, when those who had an upward trend during the later portion of their ICU stay (both uptrending and convex) were combined and compared with those with a downtrending group (downtrending and concave), the in-hospital mortality of the uptrending group was 52%, which was significantly higher than the 32% mortality rate of the downtrending group (*p*=**0.04**).

## 4. Discussion

This retrospective study investigated the association between RDW and mortality in ARDS patients and presents two main findings. First, RDW values at the time of ARDS diagnosis improved a predictive model for ICU, in-hospital, and 90-day mortality in ARDS patients. Second, the RDW trend may have prognostic value for ARDS patients during their hospital course.

While RDW as a single marker is nonspecific, adding it to a prognostic model that includes previously described variables associated with ARDS outcomes improves the model. In our analysis, we used a base model with a reasonable predictive value for ICU mortality (AUC of 76%) that included established prognostic variables in ARDS (PaO_2_/FiO_2_ ratio, age, sex, comorbidities, and SOFA score) [2, 7, 12, 14, 15]. Adding RDW to the base model improved the predictive probabilities of both ICU death for 46% of the patients using the Net Reclassification Improvement (NRI). This finding was confirmed by IDI (reclassification statistics) and an AUC improvement.

RDW is a measure that represents variations in the dimensions of circulating red blood cells that has been traditionally looked at as a marker of hematological disorders such as nutritional anemia and other processes causing destruction to red blood cells [16]. However, recent studies have investigated the use of RDW as a prognostic factor in different acute and chronic conditions including coronary artery disease, heart failure, and acute kidney injury patients and in critically ill patients [6, 7, 9, 17]. In our analysis, we investigated the association between RDW and ARDS outcomes which had not previously been reported.

Initial RDW values have been studied extensively as a prognostic marker in acute conditions, but still little is known about the prognostic value of RDW trends during a hospitalization. Others have investigated whether the difference between RDW at the time of admission and 72 hours later in severe sepsis or septic shock patients had predictive value. These results showed a significant association between an interval increase in RDW and mortality [18]. In our analysis, we reported the trend in RDW value during the full hospitalization period after excluding patients with an insufficient number of values. Regression with a linear and quadratic term for time was used to divide RDW trends into 5 categories. While most patients had no significant trend in their RDW values, in-hospital mortality for the small number of patients who had upward trends was higher than patients with downward trends, suggesting a possible prognostic value RDW trajectory in ARDS patients. However, given the small number of patients with measurable RDW trends in our study, additional research is needed to further evaluate the association between RDW trends and mortality.

It is proven that RDW can be used as a prognostic marker in other acute and chronic medical conditions, but the implementation of RDW into clinical practice remains limited despite its availability. Limitations include its moderate sensitivity and specificity when used alone [6]. The underlying mechanism of the association between elevated RDW values and poor outcomes remains unclear. Possible explanations for the association between elevated RDW and poor outcomes include systemic inflammation, as elevated RDW has been shown to correlate with interleukin-6 in one analysis [19]. Systemic inflammation can affect red blood cells maturation and bone marrow function by decreasing the production of erythropoietin (which induces erythrocyte maturation and proliferation) [20, 21]. Systemic inflammation and a reduction in erythropoietin result in both an increase in the relative number of immature, larger cells, and time needed for these cells to become matured resulting in elevations in red blood cell size variability and thus RDW. Of note, studies have demonstrated the prognostic value of RDW after adjusting for inflammatory markers [21].

Oxidative stress has also been proposed as a contributing factor to increases in RDW by reducing RBC life-span and increasing the relative number of large premature red blood cells [18, 22]. Hunziker et al. have proposed an association between RDW and physiological reserve [9]. The response to acute hypoxia and stress is different between individuals as patients with more physiological reserve have the ability to produce more mature red blood cells in shorter durations of time while under oxidative stress. Therefore, it is argued that RDW and physiological reserve are divergent [9]. Given all these possible explanations, it is reasonable to consider an elevated RDW as a sign of multiple underlying physiological processes associated with more severe illness. Additional research is warranted to fully elucidate the relevant mechanisms altering RDW as this focus of work may shed additional light upon the dominant physiological disturbances occurring during severe ARDS.

There are several limitations to our study. First, as a retrospective observational analysis, we were only able to establish a statistical relationship between elevated RDW and mortality in ARDS patients without assessing causes of mortality or underlying pathophysiological mechanism. Also, we did not have all needed variables such as erythropoietin level, CRP, or iron studies for many of the patients included. The final limitation was the number of patients whose RDW trajectory could not be categorized despite the use of sophisticated statistical analysis. The strengths of this study include the relatively large number of ARDS patients included and the availability of a rich database with extensive clinical details which allowed for the accurate identification of patients meeting the Berlin criteria for ARDS.

Improving traditional severity and prognostic measures in ARDS patients is needed, given the high mortality rate of this group. More accurate severity scores and predictive models may help physicians better assess a patient's need for more invasive therapeutic interventions and guide discussions regarding prognosis and goals of care with families. It is also of extreme importance to have a more accurate severity measure in clinical trials that investigate new modalities of treatment in ARDS. Cost-effectiveness is another advantage of utilizing RDW given that it is routinely checked in ICU patients as a part of the complete blood count at no additional expense.

Our study shows that both initial RDW values and their trajectory of change during an ICU stay may provide prognostic information in those admitted with ARDS. RDW measurements should be considered worthy of inclusion in ARDS predictive models.

## Figures and Tables

**Table 1:** Baseline characteristics based on the ICU survival status.

	Survivors (*n* = 205)	Nonsurvivors (*n* = 113)	All subjects (*n* = 318)
Age (years), mean (SD)	54.0 (17.4)	67.0 (16.8)	58.6 (18.3)
Males, *n* (%)	100 (48.8)	65 (57.5)	165 (51.9)
Females, *n* (%)	105 (51.2)	48 (42.5)	153 (48.1)
RDW, mean (SD)	14.9 (2.1)	16.0 (2.6)	15.3 (2.3)
PaO_2_/FiO_2_ ratio, mean (SD)	149.1 (72.1)	125.8 (73.5)	140.8 (73.3)
SOFA score, mean (SD)	9.5 (4.2)	11.3 (5.0)	10.1 (4.6)
Congestive heart failure, *n* (%)	59 (28.8)	42 (37.2)	101 (31.8)
Cardiac arrhythmias, *n* (%)	42 (20.5)	25 (22.1)	67 (21.1)
Pulmonary circulation disorder, *n* (%)	3 (1.5)	4 (3.5)	7 (2.2)
Hypertension, *n* (%)	53 (25.9)	33 (29.2)	86 (27.0)
Chronic pulmonary disease, *n* (%)	29 (14.2)	15 (13.3)	44 (13.8)
Diabetes, *n* (%)	37 (18.1)	20 (17.7)	57 (17.9)
Chronic kidney disease, *n* (%)	7 (3.4)	5 (4.4)	12 (3.8)

**Table 2:** Odds ratios for base model and model with RDW included.

	Base model odds ratio (95% CI)	Base model + RDW odds ratio (95% CI)
Age	1.05 (1.04, 1.07)	1.05 (1.03, 1.07)
Male	1.34 (0.80, 2.26)	1.53 (0.89, 2.62)
No. of conditions^*∗*^		
0	Reference	Reference
1	1.02 (0.51, 2.05)	1.02 (0.50, 2.07)
2	0.84 (0.37, 1.93)	0.88 (0.40, 1.88)
≥3	0.75 (0.33, 1.73)	0.84 (0.38, 1.89)
SOFA score	1.10 (1.04, 1.17)	1.08 (1.02, 1.15)
First documented PaO_2_/FiO_2_ after the time of diagnosis	0.996 (0.992, 0.999)	0.995 (0.992, 0.999)
RDW at the time of diagnosis		1.22 (1.08, 1.38)

^*∗*^Conditions included: congestive heart failure, cardiac arrhythmias, valvular disease, chronic pulmonary, hypertension, paralysis, diabetes, renal failure, and metastatic cancer.

**Table 3:** Measures of discrimination and predictive ability.

	Outcome	Category	Base model	Base model + RDW	Comparison value (95% CI)	*p*-value
AUC	ICU mortality		0.758	0.784	0.026 (0.000, 0.052)	0.048
	In-hospital mortality		0.746	0.784	0.038 (0.007, 0.069)	0.02
	90-day mortality		0.755	0.793	0.039 (0.008, 0.069)	0.01

Net reclassification improvement (NRI)				Net % improvement	NRI (95% CI)	
	ICU mortality	Died	Reference	2%	0.463 (0.238–0.687)	0.001
		Survived	Reference	44%		
	In-hospital mortality	Died	Reference	10%	0.475 (0.258, 0.692)	<0.0001
		Survived	Reference	37%		
	90-day mortality	Died	Reference	1%	0.522 (0.311–0.733)	<0.001
		Survived	Reference	51%		

Integrated discrimination improvement (IDI)			Model average prediction	Model average prediction	IDI (95% CI)	
	ICU mortality	Died	0.478	0.498	0.032 (0.010–0.054)	0.005
		Survived	0.290	0.279		
	In-hospital mortality	Died	0.541	0.571	0.054 (0.029–0.080)	<0.001
		Survived	0.363	0.339		
	90-day mortality	Died	0.556	0.588	0.060 (0.033–0.086)	<0.001
		Survived	0.365	0.339		

**Table 4:** RDW trajectory and ARDS in-hospital mortality.

Trend	Concave	Down	No trend	Convex	Up
Representative trends	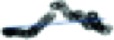	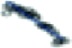	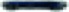	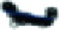	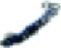
Mortality, *n* (%)	8 (31)	8 (33)	70 (40)	14 (48)	11 (58)
Total number of patients	26	24	174	29	19

## Data Availability

The data used to support the findings of this study were supplied by the Multiparameter Intelligent Monitoring in Intensive Care II (MIMIC-II) database. MIMIC-II is a publically available database of prospectively collected data for all admissions to the intensive care units at Beth Israel Deaconess Medical Center of Boston.

## Additional Points

An elevation in red cell distribution width (RDW) is associated with mortality in patients with coronary disease and kidney injury. The purpose of this study is to create a predictive model for mortality in acute respiratory distress syndrome (ARDS) using clinical factors and to determine whether the addition of RDW to the model improves its accuracy.
